# Fatal Case of Vomiting in Pregnancy, with Notes

**Published:** 1894-11

**Authors:** William L. Nolen

**Affiliations:** Chattanooga, Tenn.


### FATAL CASE OF VOMITING IN PREGNANCY, WITH NOTES.*

BY WILLIAM L. NOLEN, M.D.,

CHATTANOOGA, TENN.

In reporting this case I will not mention, much less discuss, the common remedies used for vomiting in pregnancy, for they are familiar to every member of this Society. The point I wish to bring out is to endeavor to establish the border line, where medical measures shall be given up, and the mechanical skill of the physician brought into play to empty the uterus of its contents. I know along this line one treads on holy ground, and two lives are held in our hands, and often the critical period comes when we must take, or rather destroy, one of these in order to save the other.

When such a question as this confronts us, what a vast field for contemplation arises. On the one hand, the loss of infant life, with all the vast possibilities of its yet undeveloped resources, with the inestimable good it may be to its fellow creatures. On the other, the loss of two, and no man can deny that the loss of a wife is a public as well as a private calamity.

There is only a step between the physiological and the pathological in many diseases, and as for point of time when abortion offers the only cure for pregnacious vomiting, we must admit there is to be considered no trifling question. Nothing is to be more deprecated than the induction of abortion for purely domestic reasons. I am speaking of this measure along legitimate lines. It

*Read before the Chattanooga Medical Society, August 17, 1894.

has come under the experience of many of us that some medical men are so sentimental in their religious convictions, rather than resort to this last measure, they readily sacrifice two lives instead of one. Then, again, there are those who look upon the severer form of vomiting, when from such constant irritation of the stomach it throws off its epithelium, from ignorance they assume that every thing is safe and well, when there can be no safety; and from this last cause, mainly from ignorance of the medical attendant in an adjoining State I come to report this case.

A. M., aged twenty-two, weighing one hundred and sixty pounds, became pregnant eight and a half months ago; vomiting troublesome from the beginning. What measures were used I am not able to learn. At the end of the third month vomiting increased in severity; nothing could be retained on the stomach; water was rejected as soon as swallowed; emaciation began, alimentation was nothing. This condition continued, gradually growing worse, and the epithelium was thrown off the stomach in such quantities the patient compared it to eating rags. At the end of the sixth month exhaustive diarrhea began, and this, with the vomiting, continued until at the end of the eighth month. The patient wasted from one hundred and sixty to ninety pounds. Labor came on itself, a living child was born, but lived only a few hours. The mother, soon after labor, sank into a stupor, and in a few hours death ended the scene.

What can be said of the medical man in such cases as this, when he fails to do that which he should do? There is a saying that age does not always bring wisdom, and when I hear a man proclaiming, "I have practiced medicine for over forty years, and my experience has been wonderful and wide, and there is no field wherein I have not tilled," I know this is a dangerous medical adviser.

Now the questions that must be considered are these:

What are the signals which warn us of coming danger? and when is the uterus to be emptied of its contents?

